# Association sarcoïdose et maladie de Horton: à propos d'un cas

**DOI:** 10.11604/pamj.2015.20.98.5946

**Published:** 2015-02-04

**Authors:** Sameh Marzouk, Hela Hriz, Moez Jallouli, Yosra Cherif, Zouhir Bahloul

**Affiliations:** 1 Service de Médecine Interne, CHU Hédi Chaker, Sfax, Tunisie

**Keywords:** Sarcoïdose, maladie de Horton, vascularite, sarcoidosis, Horton disease, vascularitis

## Abstract

La sarcoïdose peut être associée à d'autres maladies inflammatoires. Elle est exceptionnellement associée à une maladie de Horton posant un problème nosologique sur le caractère fortuit ou non de cette association. Nous rapportons l'observation d'une patiente, âgée de 68 ans, chez qui le diagnostic de sarcoïdose avec atteinte rénale, hépatique, oculaire, articulaire et signes généraux a été retenu et ayant été traitée par corticothérapie avec une bonne évolution. 3 ans plus tard elle a présenté des céphalées fronto-temporales associées à une claudication massétérienne et un syndrome inflammatoire biologique. La biopsie de l'artère temporale a conclu à une artérite à cellules géantes. L’évolution a été favorable sous corticothérapie. L'association d'une maladie de Horton à une sarcoïdose suggère un lien éventuel entre ces deux affections.

## Introduction

La sarcoïdose est une maladie inflammatoire multiviscérale de cause inconnue, secondaire à une réponse immunitaire incontrôlée pouvant toucher tous les organes [1]. Elle est caractérisée par une infiltration tissulaire constituée de granulomes tuberculoïdes à cellules géantes, sans nécrose caséeuse associée [2]. Son incidence annuelle est estimée entre 16 et 22 cas pour 100 000 habitants avec une légère prédominance féminine. L’évolution de la maladie est variable, caractérisée par une guérison spontanée, des poussées itératives ou l'instauration insidieuse d'une fibrose [3]. L'association d'une sarcoïdose avec une autre affection systémique dysimmunitaire est rarement décrite. Ont été rapportées des associations avec le lupus érythémateux systémique, la cirrhose biliaire primitive, la néphropathie à IgA, la sclérodermie systémique, les spondylarthropathies, les entérocolopathies inflammatoires et la maladie de takayasu [4–6]. Son association à une maladie de Horton (MH) est exceptionnelle. Nous en rapportons une nouvelle observation, qui pose un problème d'ordre nosologique sur le caractère fortuit ou non de cette association.

## Patient et observation

Mme KR âgée de 71 ans a été hospitalisée en Mars 2003 pour une fièvre nocturne associée à une asthénie, un amaigrissement non chiffré et des arthralgies inflammatoires des deux chevilles. L'examen à son admission notait un poids de 44kg pour une taille de 1,58m, une pression artérielle à 130/70mm Hg et des pouls temporaux présents et symétriques. L'examen cardio-pulmonaire, cutané et neurologique était normal. Il n'existait pas d'adénopathie ni d'organomégalie. L'examen ophtalmologique trouvait une uvéite antérieure associée à un syndrome sec. La biologie montrait une vitesse de sédimentation (VS) à 120 mm à la première heure, une C-réactive protéine (CRP) à 50mg/l, une hypergammaglobulinémie à 19g/l,une lymphopénie à 1200 E/mm^3^,des plaquettes à 455000E/mm^3^ et un taux d'hémoglobine à 13g/dl. Il existait une insuffisance rénale avec une créatinémie à 170 µmol/l sans anomalie des sédiments urinaires et une cholestase hépatique (gamma GT à 4N, phosphatase alcaline à 2N). La calcémie et la calciurie étaient normales. Le bilan immunologique était négatif (recherche de facteurs rhumatoïdes, d'anticorps antinucléaires, d'anticorps anticytoplasme des polynucléaires neutrophiles et d'anticorps antimithochondries). Le taux sérique de l'enzyme de conversion de l'angiotensine était élevé (100 UI/L (n < 60). Les sérologies des hépatites virales B et C étaient négatives. L'intradermoréaction trouvait une anergie tuberculinique. « La recherche de BK dans les crachats, les urines et le tubage gastrique (examen direct et cultures) était négative. La radiographie pulmonaire, l’électrocardiogramme, les explorations fonctionnelles respiratoires, l’échographie abdomino-pelvienne, le myélogramme et la biopsie de l'artère temporale étaient normaux. La biopsie des glandes salivaires accessoires a conclu à une sialadénite granulomateuse. Au terme des explorations, le diagnostic de sarcoïdose avec atteinte rénale hépatique, oculaire, articulaire et signes généraux a été retenu et la patiente a été traitée par une corticothérapie à raison de 1mg/kg/j entrainant une amélioration clinique et biologique (régression du syndrome inflammatoire, normalisation de bilan hépatique et de la créatinine sanguine). La corticothérapie a été arrêtée au bout de 2 ans. Une année plus tard, la patiente a été hospitalisée de nouveau pour des céphalées fronto-temporales pulsatiles et insomniantes associées à une claudication massétérienne et à un amaigrissement de 2 kg évoluant depuis deux semaines. A l'admission, elle était apyrétique, les pouls temporaux étaient faibles. L'examen ophtalmologique était normal. Le reste de l'examen était sans particularité. A la biologie, il existait un syndrome inflammatoire avec une VS à 70 mmH1 et une CRP à 60mg/l, des phosphatases alcalines à 1,5N. Le reste du bilan était normal. La radiographie du thorax était normale. La biopsie de l'artère temporale a mis en évidence une artérite temporale à cellule géante. Le diagnostic de maladie de Horton a été retenu et la patiente a été mise de nouveau sous corticothérapie (1mg/kg/j) maintenue pendant un mois avec une bonne évolution clinique et biologique. Le recul actuel est de 6 ans.

## Discussion

La sarcoïdose est une affection systémique d’étiologie inconnue, touchant avec prédilection l'appareil respiratoire et les voies lymphatiques, et caractérisée par la formation de granulomes immunitaires dans les organes atteints [2]. Les formes extrathoraciques isolées sont plus rares, soit environ 20% des cas [7]. Les manifestations vasculaires de la sarcoïdose sont exceptionnelles [8]. Elles sont surtout attribuées à une compression extrinsèque des artères et des veines par l'atteinte ganglionnaire. La sarcoïdose affecte préférentiellement les vaisseaux de gros calibre chez les sujets africains américains, alors que l'atteinte des petits vaisseaux concerne surtout les sujets de peau blanche. La signification des atteintes vasculaires de la sarcoïdose est discutée: atteintes spécifiques ou association à d'autres maladies [9]. La coexistence de la sarcoïdose avec d'autres pathologies systémiques notamment à des vascularites est rare. Ahuja et al ont rapporté un cas de sarcoïdose ayant été précédé par une granulomatose de Wegener [10]. Quelques cas de vascularites des gros troncs ont été également rapportés. La maladie de Horton est une artérite inflammatoire touchant les artères de gros et moyen calibre avec une prédilection pour les branches crâniennes des artères provenant de l'arc aortique et notamment de la carotide externe, ainsi que pour les artères à destinée ophtalmique. Elle peut cependant toucher l'aorte et ses grosses branches et plus rarement des artères viscérales diverses ou des artères des membres [11]. Elle est exceptionnellement associée à la sarcoïdose. A notre connaissance, le premier cas étant rapporté par Kinmont et al [12]. Il s'agissait d'un homme âgé de 79 ans ayant présenté une sarcoïdose 8 mois après le diagnostic d'une MH. Marcussen et al, ont découvert à l'autopsie d'un homme d’âge moyen, une vascularite disséminée à cellules géantes des vaisseaux de gros et de moyens calibre, associée à une granulomatose vasculaire et ganglionnaire [13]. Procter et al ont également décrit le cas d'un homme âgé de 73 ans chez qui le diagnostic de sarcoïdose et de MH était concomitant [14]. Dans une revue de la littérature, et en essayant de revoir les vascularites associées à la sarcoïdose, Weiler et al, ont recensés 8 cas dominés par la maladie de Takayasu. Il n'existait aucun cas de MH [15]. De même Liozon et al [16], dans leur série comportant 250 cas de MH, la sarcoïdose n’était présente que dans un seul cas (0,4%). Il s'agissait d'un homme âgé de 67 ans chez qui la sarcoïdose était survenue 5 ans après la MH.

Notre cas présenté décrit une patiente chez qui le diagnostic de sarcoïdose a précédé de 3 ans la MH. Le diagnostic de MH a été retenu sur des éléments cliniques et histologiques. L'association d'une MH et d'une sarcoïdose a l'intérêt de soulever un certain nombre de questions sur le lien éventuel entre ces deux affections. L'atteinte vasculaire ou l'angéite granulomateuse est-elle une manifestation propre de la sarcoïdose ou c'est une réelle association. La sarcoïdose a été déjà décrite à d'autres maladies granulomateuses comme la maladie de Takayasu et la maladie de Crohn [6]. De même ces 2 maladies paraissent partager des similitudes étiopathogéniques. En effet, une susceptibilité génétique commune, notamment l'association avec le groupe HLA DR4, des anomalies de la réponse immunologique comme la réponse immune de type Th1 et l'hypothèse infectieuse été ont évoquée dans l’étiopathogénie des deux maladies [1].

## Conclusion

Bien que l'association de la sarcoïdose à une MH soit exceptionnelle, elle pourrait ne pas être fortuite. Cette association pourrait relever d'un mécanisme étiopathogénique commun aux deux maladies.
